# Reprogrammable reflection-transmission integrated coding metasurface for real-time terahertz wavefront manipulations in full-space

**DOI:** 10.1038/s41598-024-61638-7

**Published:** 2024-05-15

**Authors:** Parsa Farzin, Amir Saman Nooramin, Mohammad Soleimani

**Affiliations:** School of Electrical Engineering, Iran University of Science and Technology, Tehran, 1684613114 Iran

**Keywords:** Applied optics, Optical materials and structures, Optical physics, Optical techniques, Other photonics

## Abstract

In recent years, there has been notable advancement in programmable metasurfaces, primarily attributed to their cost-effectiveness and capacity to manipulate electromagnetic (EM) waves. Nevertheless, a significant limitation of numerous available metasurfaces is their capability to influence wavefronts only in reflection mode or transmission mode, thus catering to only half of the spatial coverage. To the best of our knowledge and for the first time, a novel graphene-assisted reprogrammable metasurface that offers the unprecedented capability to independently and concurrently manipulate EM waves within both half-spaces has been introduced in the THz frequency band. This intelligent programmable metasurface achieves wavefront control in reflection mode, transmission mode, and the concurrent reflection-transmission mode, all within the same polarization and frequency channel. The meta-atom is constructed with two graphene sections, enabling straightforward modification of wave behavior by adjusting the chemical potential distribution within each graphene segment via an external electronic source. The proposed functionalities encompass various programmable modes, including single and dual beam control in reflection mode, dual beam control in transmission mode, simultaneous control of dual beams in reflection mode-direct transmission, and vice versa, and control of beam steering in reflection mode-dual beams in transmission mode simultaneously. The proposed metasurface is expected to be reprogrammable due to wavefront manipulation in both half-spaces separately and continuously for various applications such as imaging systems, encryption, miniaturized systems, and next-generation wireless intelligent communications.

## Introduction

In recent years, the scope of Terahertz (THz) science and technologies has reached maturity and attracted massive attention due to their potential applications in biomedicine, wireless communication, and imaging^1–4^. Efforts to fully harness the benefits of THz waves have spurred significant advancements in modern and multifunctional THz devices. In the past decade, metamaterials have attracted much attention in scientific research and engineering fields, especially in the THz frequency band, due to their unusual and tunable electromagnetic (EM) waves properties that are not attainable in natural material^5,6^. Metamaterials, typically composed of artificially periodic or quasi-periodic structures with sub-wavelength scales, offer a new design strategy for functional materials, leading to exotic phenomena and applications such as negative refraction^7^, zero refractive index^8^, perfect absorption^9^, and cloaking^10^. However, metamaterials encounter significant practical constraints due to lossy characteristics, the strong dispersion of resonant responses, and the manufacturing complexities of three-dimensional bulky structures^11^. As an alternative, two-dimensional (2D) counterparts of metamaterials, known as metasurfaces (MSs), have been intensively investigated due to their promising advantages, including compactness, low cost, high surface integrity, and ease of fabrication^12^. They thereby overcome the challenges encountered by their 3D counterparts^13^. As an emerging platform, MSs are inspiring a flourishing of research interest with exceptional manipulation of EM wave amplitude^14,15^, phase^16–19^, and polarization^20,21^. In this regard, numerous MSs have been developed to achieve extraordinary applications, such as meta-lenses^22^, orbital angular momentum generation (OAM)^23^, holography^24,25^, and cloaking^26^. As one of the most promising candidates, Cui et al. have recently introduced the concept of digital MSs, revolutionizing the field by linking the physical and digital worlds. This innovative approach enables a fresh perspective on MSs from the standpoint of information science^27^. Unlike conventional MSs, coding MSs with well-defined elements can be digitalized and programmed using a field-programmable gate array (FPGA). By controlling sequences of digital coding states “0” and “1” with opposite-phase responses (0^∘^ and 180^∘^), the MSs allow for the manipulation of EM waves with various functionalities^28^. Moreover, this coding and programmable MSs facilitate dynamic and real-time control of EM waves, offering the extraordinary potential to establish dynamic MSs that can be used in applications such as wireless communications in the microwave^29^, THz^30^, and light-to-microwave^31,32^. The emergence of multifunctional MSs structures has attracted considerable attention in recent years. Unlike previous single-functional MS, these versatile MSs can now achieve a diverse range of electromagnetic functions simultaneously^33^. Despite the great achievements attained so far, we note that the wave manipulation capabilities of multifunctional MSs have been much less explored^34^. Moreover, the manipulation of EM waves by these MSs is usually limited to half-space, and most devices can only operate in reflection^35,36^ or transmission mode^37^. For instance, Zhang et al.^38^ successfully manipulated the reflected wave for both x- and y- polarizations of the beam independently. Consequently, they can only manipulate the reflected wave, leaving half-space unutilized.^39^. This limitation significantly hinders their potential applications. While some MSs studies have proposed to manipulate the wavefront of both the reflected and transmitted waves in the full space. In^40^, Pan et al. MS designed in the microwave band achieves remarkable EM wave control for both reflection and transmission modes through dimension adjustments, effectively operating in two frequency bands: reflecting x-polarization and transmitting y-polarization in the $f_1$ frequency band, also transmitting x-polarization in the $f_2$ frequency band. Phase control is also achieved by changing dimensions, enabling precise manipulation of the EM waves. The reflected and transmitted waves are manipulated using orthogonal grating-wire layers, thus allowing for control over the EM waves. In another study, described in^41^, Wu et al. researchers demonstrated MSs operating in the microwave band that effectively controls y-polarized waves for both reflected and transmitted wave modes by adjusting the bias of the diode pin. Notably, phase control is achieved through dimension changes of the patch, enabling wave manipulation in both reflection and transmission modes. However, a fundamental issue with the structures introduced is their inability to be reprogrammed, resulting in a fixed performance post-construction, limiting their versatility across various applications. Finally, in^42,43^, Cui et al. and Bao et al. proposed a structure operating in the microwave band, which utilizes pin diodes to control not only the reflected and transmitted EM waves but also the phase. This design allows real-time manipulation of waves in full space. However, as the frequency increases from microwave to terahertz, the availability of PIN diodes becomes limited, making their use impractical^44^. Consequently, achieving reprogrammable THz metamaterials that match the performance of existing microwave materials poses a significant challenge. Researchers to overcome this limitation have turned to active materials such as graphene^45,46^, vanadium dioxide ($VO_2$)^47,48^, InSb^49^, and liquid crystals^50^ to manipulate THz EM waves. However, achieving control of reflected and transmitted waves in MSs is not limited to the microwave band, and efforts have also been made in the THz band. In^51^, Dong et al. the proposed structure achieves control of the reflection and transmission of EM waves in two different frequency bands through the use of InSb temperature changes. Additionally, phase control is obtained by adjusting the dimensions of the structure. To the best of the author’s knowledge, all the research in the THz band that has been done on manipulation of full-waves in an MS involves at least one task of controlling the amplitude (reflection or transmission) or the phase of the EM wave by changing the dimensions^52–56^. Therefore, in all of these strategies, MSs are designed for a specific application, and once fabricated, their performance remains constant. Thus, metasurfaces capable of real-time control EM waves in both reflection and transmission modes are highly beneficial in 6G and intelligent omni surface (IOS) wireless communication applications.

Graphene, a flat monolayer of carbon atoms organized in a two-dimensional (2D) honeycomb-like lattice, has garnered significant attention worldwide due to its exceptional electrical and mechanical properties and the design freedom it offers^57,58^. The ability to arbitrarily control the electrical properties of graphene through external biasing has opened up possibilities for the development of radically different photonic devices^59^. By harnessing this distinctive property of graphene, it becomes possible to obtain distinct amplitude and phase responses, leading to diverse capabilities, including manipulating wavefront^60^, polarization converters^61–63^, and tunable absorption^64^.

Although we have recently introduced a graphene-based metasurface that enables control over reflection, transmission, absorption, and polarization conversion for both reflection and transmission modes, our current design is restricted by its lack of phase control, limiting its manipulation of the wavefront across two half-spaces^65^. To address these constraints, For the first time to the best of our knowledge in the THz frequency band, we introduce an intelligent reprogrammable graphene-assisted metasurface that holds the capability to independently and concurrently manipulate EM wavefront across both half-spaces in real-time at the same polarization and frequency channel. The meta-atom is constructed with two graphene sections, enabling straightforward modification of wave behavior by adjusting the chemical potential distribution within each graphene segment via an external electronic source by FPGA. Through the utilization of two graphene sections within the meta-atom, independent or simultaneous control of reflection, transmission, and phase adjustments can be achieved across the entire space in real-time. By using two graphene sections integrated in the meta-atom, it becomes feasible to independently or concurrently manipulate reflection, transmission, and phase characteristics throughout the full-space in real-time. To assess the efficacy of the proposed metasurface, we explore diverse functionalities in both reflection and transmission modes, both individually and concurrently. These include single and dual beam control in reflection mode, dual beam control in transmission mode, simultaneous control of dual beams in reflection mode-direct transmission, and vice versa, and dynamic control of a single beam in reflection mode-dual beams in transmission mode, all within the same polarization and frequency channel. We firmly believe that the introduced metasurface elevates the landscape of intelligent multifunctional metasurfaces and carries substantial potential across a range of applications, including optical communication, imaging systems, and next-generation wireless intelligent communications.

**Figure 1:**
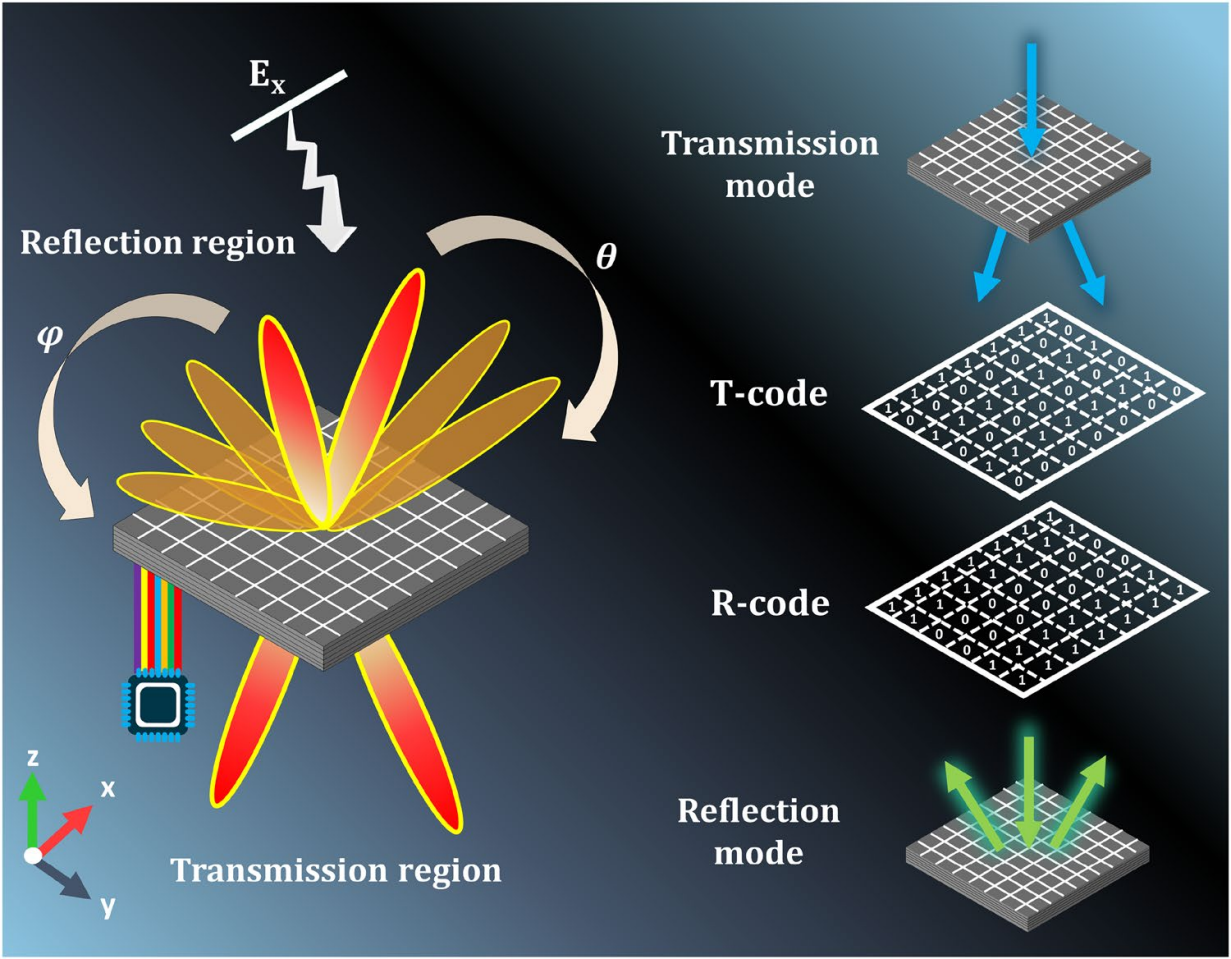
Imagine a versatile and programmable metasurface concept, composed of an array of meta-atoms capable of dynamically altering their operational states to serve different functions. This intelligent and adaptable metasurface can seamlessly transition between reflection, transmission, and simultaneous reflection-transmission, all achieved through the independent modulation of two distinct graphene sections by an integrated external FPGA-based controller. Through the utilization of unique preconfigured coding sequences within each of graphene sections, this intelligent metasurface can swiftly execute a diverse range of functions in real-time.

## Materials and methods

### Complex graphene’s surface conductivity

Graphene, a 2D material, consists of carbon atoms arranged in a hexagonal lattice, garnering widespread attention in the last decade as a zero-gap semiconductor with unique electrical, thermal, optical, and mechanical properties. Furthermore, owing to its boundary conditions, graphene’s extreme sensitivity to external stimuli makes it an excellent candidate for manipulating THz waves. Through the Hall Effect, the AC conductivity of graphene can be tuned using external electrostatic or magnetostatic bias. Moreover, due to its mono-atomic structure, graphene can be locally represented by a complex surface conductivity tensor^66,67^:

1 $$\begin{aligned} \sigma (\omega _c, \mu (E_0 ), \Gamma , T, B_0 )= {\hat{x}}{\hat{x}}\sigma _{xx} + {\hat{x}}{\hat{y}}\sigma _{xy} + {\hat{y}}{\hat{x}}\sigma _{yx} + {\hat{y}}{\hat{y}}\sigma _{yy} \end{aligned}$$

where $\omega$ is the radian frequency, $\mu _c$ is the chemical potential, $\Gamma =1/2\tau$ is the phenomenological scattering rate with $\tau$ being the electron-phonon relaxation time. T is the room temperature, $E_0$ and $B_0$ are electrostatic and magnetostatic bias fields, respectively. Without magnetostatic bias, the off-diagonal components of the surface conductivity tensor become vanish, resulting in the notice of isotropic behaviors in the graphene monolayer^66,67^. The graphene monolayer can be represented electrically as an infinitely thin conducting layer with a surface resistance $R_g$ characterized by a complex-valued conductivity surface. According to the Kubo formula, the complex surface conductivity of graphene can be expressed with the help of interband and intraband transitions^66,68^.

2 $$\begin{aligned} R_g = 1/\sigma _s = 1/(\sigma ^s_{intra}+\sigma ^s_{inter}) \end{aligned}$$

3 $$\begin{aligned} \sigma ^s_{intraband} (\omega ) = -\frac{ie^2 k_B T}{\pi \hbar ^2 (\omega -i/\tau )} \left[ \frac{\mu _c}{k_B T} + 2ln(e^\frac{-\mu _c}{k_B T}+1)\right] \end{aligned}$$

4 $$\begin{aligned} \sigma ^s_{intraband} (\omega ) = -\frac{ie^2}{4\pi \hbar } ln\left[ \frac{2|\mu _c |-(\omega -i/\tau )\hbar }{2|\mu _c |+(\omega -i/\tau )\hbar }\right] \end{aligned}$$

Here, e is electron charge, $\hbar$ represents the reduced Plack’s constant, $\mu _c$ is the chemical potential, $k_B$ is the Boltzmann’s constant. At room temperature and low terahertz frequency, interband transitions can be neglected due to the Pauli Exclusion Principle, as the photon energy is $\hbar \omega \ll E_f$^69^. In our simulations, we considered two parameters of temperature and relaxation time equal to T= 300 K and $\tau$=1 ps, respectively, which are kept constant throughout this study. Additionally, the relative permittivity of graphene layers is expressed as $\epsilon _rG = 1 + \sigma _s/(j\omega \sigma _0 \Delta )$, where $\Delta$ denotes the ultra-thin thickness of the graphene layer. It is worth mentioning that the surface conductivity of the graphene layer can be manipulated through an external electrical bias. By changing the chemical potential of graphene, the properties of graphene can be tuned between dielectric and conductive states. Increasing the chemical potential of graphene causes it to change from dielectric to conductor and vice versa^70^. As a result, it enables real-time dynamic switching for all the different functions considered for the MS designed can be changed through an external electrical bias in this paper. Detailed information can be found in Supplementary Information A.

### Design principle

Currently, most reconfigurable full-space metasurfaces are limited to dynamic control of only one EM property, (such as phases or reflection-transmission mode of operation). In addition, achieving full-space multifunctional capabilities with these metasurfaces are also limited to different frequency/polarization channels. One of the major significant drawbacks of single-layer metasurfaces is their limited interactions with incident EM waves, resulting in restricted EM responses^71^. Thus, the potentially restricted phase modulation observed in a single graphene layer can be overcome through the implementation of a stacked structure. In this configuration, each layer effectively contributes to a wider range of phase tuning capabilities^72,73^. Accordingly, several layers are needed to achieve a metasurface with different functions. To achieve real-time manipulation of the wavefront in full space, within the same frequency and polarization channel, a cascaded multilayer structure integrated with graphene is utilized. By dynamically adjusting the chemical potential of graphene, we can effectively control the transmission-reflection operation mode and phase responses in real-time. What sets this structure apart is that all of these functions are performed in real-time, facilitated by the integration of graphene.

**Figure 2:**
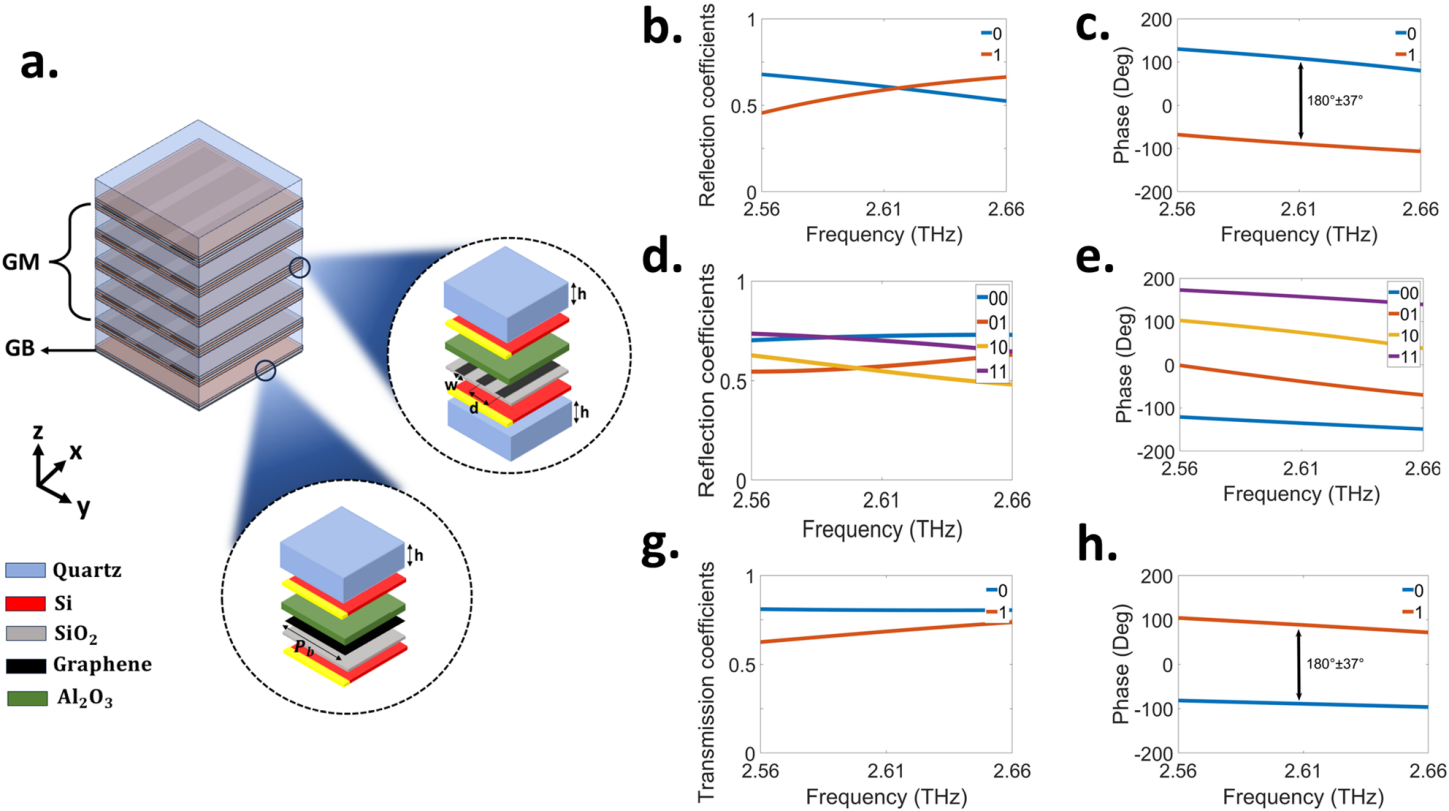
(**a**) Programmable meta-atom geometry. The meta-atom comprises two sections of graphene denoted as G1 and G2. Each section is constructed with a layered structure of Si/SiO_2_/Graphene/Al_2_O_3_/Si. (**b** and **g**) Simulated amplitude for reflection/transmission in 1-bit mode. (**c** and **h**) reflection/transmission phase in 1-bit mode. reflection (**d**) amplitude and (**e**) phase in 2-bit mode.

Figure1 shows a general schematic of the reprogrammable metasurface designed for the manipulation of EM waves on both sides of space. The proposed metasurface is composed of two distinct sections of graphene, each individually assigned to control specific functions. These two sections include reflection-transmission control section, and phase control section. As shown in Fig. 1, By applying the appropriate bias voltage to each of the graphene parts by field-programmable gate array (FPGA), the metasurface can flexibly switch between reflection mode, transmission mode, simultaneous control mode of reflection and transmission symmetrically and asymmetrically. The functionalities of the metasurface can be dynamically controlled in real-time by adjusting the chemical potential of graphene. This real-time manipulation of the full-space occurs within the same frequency band and polarization, offering versatile and instantaneous control over the metasurface’s operations. The proposed intelligent metasurface can control the reflection and/or transmission along with the manipulation of the wavefront by changing the chemical potential of the graphene layers in the backward half-space and/or forward half-space. Amplitude control is achieved by electric bias manipulation of the reflection-transmission layer, while wavefront manipulation is carried out through the phase control layer. It is worth noting that by placing reflective and transmission meta-atoms adjacently with appropriate and similar distribution, the metasurface can simultaneously control both reflection and transmission. Furthermore, by adjusting the chemical potential of the phase control layer, we can manipulate the wavefront in the forward and backward wave scatterings in the same or different ways. Controlling each characteristic of the EM wave through the design of separate layers in a metasurface not only simplifies the bias network but also grants simultaneous access to different functions. As depicted in Fig.2a, The meta-atom design includes three key components: graphene, quartz, and floating gates. Graphene components are structured into two distinct parts: the initial square-shaped graphene section (G1) with a length of $P_b$ = 49 $\upmu$m and the subsequent multi-layered graphene segment (G2) comprising w = 5 $\upmu$m wide ribbons sandwiched between quartz and floating gates. The layers within G2 consist of three graphene ribbons spaced at d = 10 $\upmu$m intervals along the y-axis. The structure further comprises six layers of quartz, each with a uniform thickness of h = 12.5 $\upmu$m and possessing a relative permittivity ($\epsilon _r$) of 3.75 and a loss tangent (tand) of 0.0004. The periodicity of each meta-atom is P = 50 $\upmu$m (The effect of the number of layers on the amplitude and phase of the reflected and transmitted wave is given in Supplementary Information B). Additionally, the floating gate is composed of polycrystalline silicon, alumina, and graphene. This combination enables the modulation of charge density within the graphene component, thus providing tunability to the structure (Further details are available in supplementary material section A). The metasurface is divided into two distinct parts, and each graphene section serves a specific function. The first part, labeled G1 and positioned at the structure’s end, controls reflection-transmission. In the middle of the metasurface, denoted as G2 and the second part is devoted to precise phase control, utilizing multiple layers of graphene. In the first section (G1), by increasing (decreasing) the chemical potential of graphene, its properties can be changed from a dielectric to a conductor (or vice versa), allowing for precise control over the incident wave^70^. When graphene is in the conductor state, the structure exhibits a reflective mode. Conversely, by reducing the external electric bias and converting graphene to a dielectric, the structure switches to a transmission state. The second section (G2) utilizes five layers of graphene nanoribbons to control the phase of reflected and transmitted waves. In this section, the chemical potential of the layers changes simultaneously, yielding different phase responses. By applying appropriate chemical potentials, 1-bit, and 2-bit phase shifts can be achieved in reflection and transmission modes, respectively. The graphene layers employed in this paper have the same dimensions, but have different chemical potentials. For the purpose of modifying the charge density within each graphene layer, we employ floating gate structures, as depicted in Fig.2. These structures consist of Si, SiO_2_, Al_2_O_3_, and single-layer graphene. Upon applying a positive bias voltage to the upper Si layer, electrons from the lower Si layer can tunnel through the SiO_2_ layer and become captured by the graphene, resulting in a heightened charge density within the graphene layer.Conversely, upon applying a reverse bias voltage to the upper Si layer, electrons from the graphene layer can tunnel through the SiO_2_ and become captured by the lower Si layer, leading to a decrease in the charge density of the graphene layer^74,75^. Furthermore, thanks to the electrical isolation of the graphene layer from the Si layers, once the external bias voltage is removed, the charge density of the graphene can remain stable over an extended duration. Consequently, there is no requirement for any additional force to maintain the constancy of the graphene charge density. It’s worth emphasizing that electron tunneling is exclusively achievable through the SiO_2_ layer; tunneling through the Al_2_O_3_ layer is rendered impractical due to the substantial thickness of Al_2_O_3_ compared to SiO_2_ (Al_2_O_3_ = 20 nm and SiO_2_ = 10 nm). The thin layers within the floating gates play a pivotal role in DC bias design. Because their thickness is significantly smaller than the operating wavelength, their influence on the amplitude and phase of the reflected and transmitted waves can be neglected. They can be fruitfully represented by a physical equivalent circuit in which each patterned graphene between two stratified media (air-quartz or quartz-quartz) is represented as an R–L–C branch (see Supplementary Information C). The supporting substrates can be conceptualized as separate transmission lines, each possessing its own intrinsic impedance and propagation constant, denoted as $\eta _c$=$\eta _0$/$\sqrt{\epsilon _r}$ and $\gamma$ = $\omega \sqrt{\epsilon }_r/c$, respectively. Here, $\eta _0$ and c refer to the characteristic impedance of free space and speed of light. Utilizing the circuit model, we calculated the amplitude and phase responses for $R_{01}$, $T_0$. The determined values for R, L, and C are presented in Supplementary Table S1 for various scenarios. Supplementary Fig. S5a,b compares the spectra obtained from the circuit model with the spectra from full-wave numerical simulations, demonstrating a excellent agreement in both amplitude and phase responses. These trends are physically understandable and can be thoroughly explained by examining the changes in the real and imaginary parts of graphene conductivity, along with considering the electrostatic capacitive coupling between adjacent elements^76^. Consequently, the development of the circuit model enhances the proposed metasurface design process, and the resulting outcomes closely align with our expectations.

**Table 1:** The essential chemical potential for each segment of the graphene meta-atom to govern the wavefront in both reflection and transmission modes.

State	G1 (eV)	G2 (eV)				
		0^∘^	90^∘^	180^∘^	270^∘^	
Reflection	1.5	0.5		1.4		1-bit
	1.5	1.1	0.15 eV	0.7	0.4 eV	2-bit
Transmission	0	0		1.5		1-bit

## Results and discussion

### control of full space

#### The scattering properties of metasurface

Introducing abrupt phase shifts through coding metasurfaces opens a new avenue for manipulating scattering patterns. Such coded metasurfaces have the ability to reflect or transmit incident waves into anomalous directions, governed by the principles of generalized Snell’s law^77^. At the outset, we examine a simple form of coding metasurfaces, characterized by a coding pattern comprising solely two interchangeable coding elements, “0” and “1,” yielding opposite reflection phase responses. In order to minimize the EM coupling between adjacent meta-atoms, the proposed metasurface is constructed from M$\times$M unit-cells, that form the so-called super unit cells or lattices. The length of each lattice is ($D_x = D_y = D$), which is equal to PM. Due to the one-to-one connection between different coding patterns of metasurface and their far-field patterns, under normal incidence, the proposed metasurface far-field scattering pattern function can be expressed by^78,79^.

5 $$\begin{aligned} F(\theta ,\phi )=f_{m,n} (\theta ,\phi ) \sum _{m=0}^{M-1} \sum _{n=0}^{M-1} A_{m,n} exp(u)\times exp(v) \end{aligned}$$

Where $u=i m k_0 D sin\theta cos\phi$, $v=i n k_0 D sin\theta sin\phi$, $f_{(m,n)} (\theta ,\phi )$ is the pattern function of lattice, $A_{(m,n)}= a_{m,n} exp (i\phi _{m,n})$ is the complex reflection coefficient, $k_0$ is the free-space wavevector, and $\theta$ and $\phi$ are elevation and azimuth angles. Due to the metasurface units being significantly smaller than the wavelength, during the calculation of the far-field pattern, we can confidently disregard $f_{(m,n)} (\theta ,\phi )$. This formula proves advantageous for predicting scattering patterns arising from different coding sequences. Nonetheless, augmenting the overall number of lattices within the metasurface leads to extended computation durations. To circumvent this limitation, a viable solution involves employing a two-dimensional inverse fast Fourier transform (2D-IFFT) from Eq. 5, resulting in a considerable acceleration of the calculations^23,80^.

#### Graphene-Based Coding design and its circuital representation

The simulations performed for each meta-particle were performed using the full-wave commercial software, CST Microwave Studio. Periodic boundary conditions are applied in the x- and y-directions while Floquet ports are also assigned to the z-direction. The meta-atoms within the proposed graphene-based metasurface are divided into two distinct components. By tuning the chemical potential of graphene in the first (G1) and second (G2) layers, control of reflected and transmitted waves is attainable real-time, along with manipulation of the EM wavefront. For reflection and transmission modes, control of the phase layer enables the attainment of 1-bit/2-bit and 1-bit metasurface, respectively. In the reflection mode, the chemical potential of G1 is equal to 1.5 eV, causing graphene to behave as a conductor. As a result, the incident wave is reflected through G1. While in the transmission mode, the value of the chemical potential of graphene G1 should be such that graphene acts like a dielectric so that the incident wave passes through the metasurface, so we consider its value to be 0 eV. In both the reflection and transmission modes, wavefront manipulation is achieved by applying appropriate chemical potentials to the graphenes within the second segment (G2). In the reflection mode, the range of graphene’s chemical potential within the middle section is adjusted from 0 to 1.4 eV to achieve a 1-bit/2-bit phase shift. Conversely, in the transmission mode, the chemical potential is varied within the same section from 0 to 1.5 eV to attain a 1-bit phase shift. It’s important to highlight that the consideration of a chemical potential exceeding 1.2 is grounded in prior research findings^46,81–83^. The chemical potentials applied to the two sections of graphene meta-atoms are denoted as A/B. Specifically, A represents the chemical potential of the first layer (G1) and B corresponds to the chemical potential of the second layer (G2). The comprehensive reflection and transmission spectra, obtained under x-polarized illumination, are illustrated in Fig.2b–h for various sets of chemical potentials applied to the first and second layers. Illustrated in Fig.2b,c shows, the selection of chemical potentials at 1.5 eV/ 0.5 eV and 1.5 eV/ 1.4 eV yields phase responses with a tuned 180-degree phase shift. Remarkably, within the frequency range of 2.56–2.66 THz, these responses effectively enable the representation of binary “0” and “1” through a 1-bit encoding scheme. Fig.2d,e shows the amplitude and phase coefficients for chemical potentials of 1.5 eV/ 1.1 eV, 1.5 eV/ 0.15 eV, 1.5 eV/ 0.7 eV, 1.5 eV/ 0.4 eV. Phase responses provide a phase shift of 90^∘^ from each other for the frequency band 2.55–2.65 THz, which can mimic the 2-bit encoding “00”, “01”, “10”, and “11”, respectively. Similarly, Fig.2 (d) and (e) shows the amplitude and phase transmission coefficients for chemical potentials 0 eV/ 0 eV and 0 eV/ 1.5 eV. The phase responses provide a phase shift of 180^∘^ from each other for the same frequency band (2.55–2.65 THz), which can mimic 1-bit encoding “0” and “1”, respectively. The key point is that all meta-atoms coded in two reflection and transmission modes have high reflection and transmission coefficients in the operational bandwidth because they operate far from their resonance frequency. It’s worth highlighting that the reflection and transmission phase responses demonstrate a nearly linear trend, a crucial aspect in the design of multi-bit coding meta-atoms. Figure 2 illustrates that the reflection/transmission coefficients of the majority of coding states are below 0.8. This is because in the frequency range of 0.1–10 THz, graphene exhibits losses due to intra-band free-carrier absorption^84^. Therefore, in a multilayered structure, losses increase. It’s worth noting that by improving the fabrication process of graphene, the losses associated with graphene will be reduced. The summary of the above discussion can be found in Table 1. Finally, we compared our purpose metasurface with other full-space metasurfaces. As demonstrated in Table 2, the previously reported metasurfaces were not capable of controlling real-time reflection, transmission, and phase for full space at THz frequencies.

**Table 2:** Comparison of the proposed metasurface with some recent reported full-space metasurface.

Refs.	Frequency	Real-time tunable reflection and transmission	Real-time tunable phase	Active element	Same frequency	Same polarization
^40^	GHz	No	No	–	No	No
^41^	GHz	Yes	No	PIN diode	Yes	Yes
^42,43^	GHz	Yes	Yes	PIN diode	Yes	Yes
^52,53,56^	THz	No	No	-	No	No
^44,51,54,60^	THz	Yes	No	Vo2	Yes	Yes
This work	THz	Yes	Yes	Graphene	Yes	Yes

#### Performance of the reconfigurable metasurface in 1-bit reflection and 1-bit transmission modes

As previously mentioned, applying distinct electric field bias values at various locations across the proposed metasurface leads to a switchable coding layout that enables real-time manipulation of EM waves. The intelligent metasurface, comprising 20 $\times$ 20 meta-atoms, is designed to govern EM wavefronts on both sides of space. When exposed to a normal x-polarized plane wave propagating along the z-direction, the metasurface reveals the corresponding far-field patterns at f = 2.61 THz. First, we set the metasurface in reflective mode, which can be equivalently regarded as a reconfigurable reflective metasurface to cover the backward half-space well. In this case, the chemical potential of the reflection-transmission layer graphene is equal to 1.5 eV. It is worth mentioning that abrupt phase changes along the x-direction cause the scattered wave to split into two symmetrical reflection beams and exhibit anomalous reflection behavior^78^. Consequently, dynamic twin-beam production can be achieved by modulating the phase layer’s chemical potential and employing stripe coding patterns with a phase difference of 180^∘^ consisting of “0” and “1” coding modes. Here, we denote these two states as the coding bites “$R_{0}$” and “$R_{1}$”. The angle direction of the twin beams can be theoretically predicted according to the generalized Snell’s law as follows^85^.

**Figure 3:**
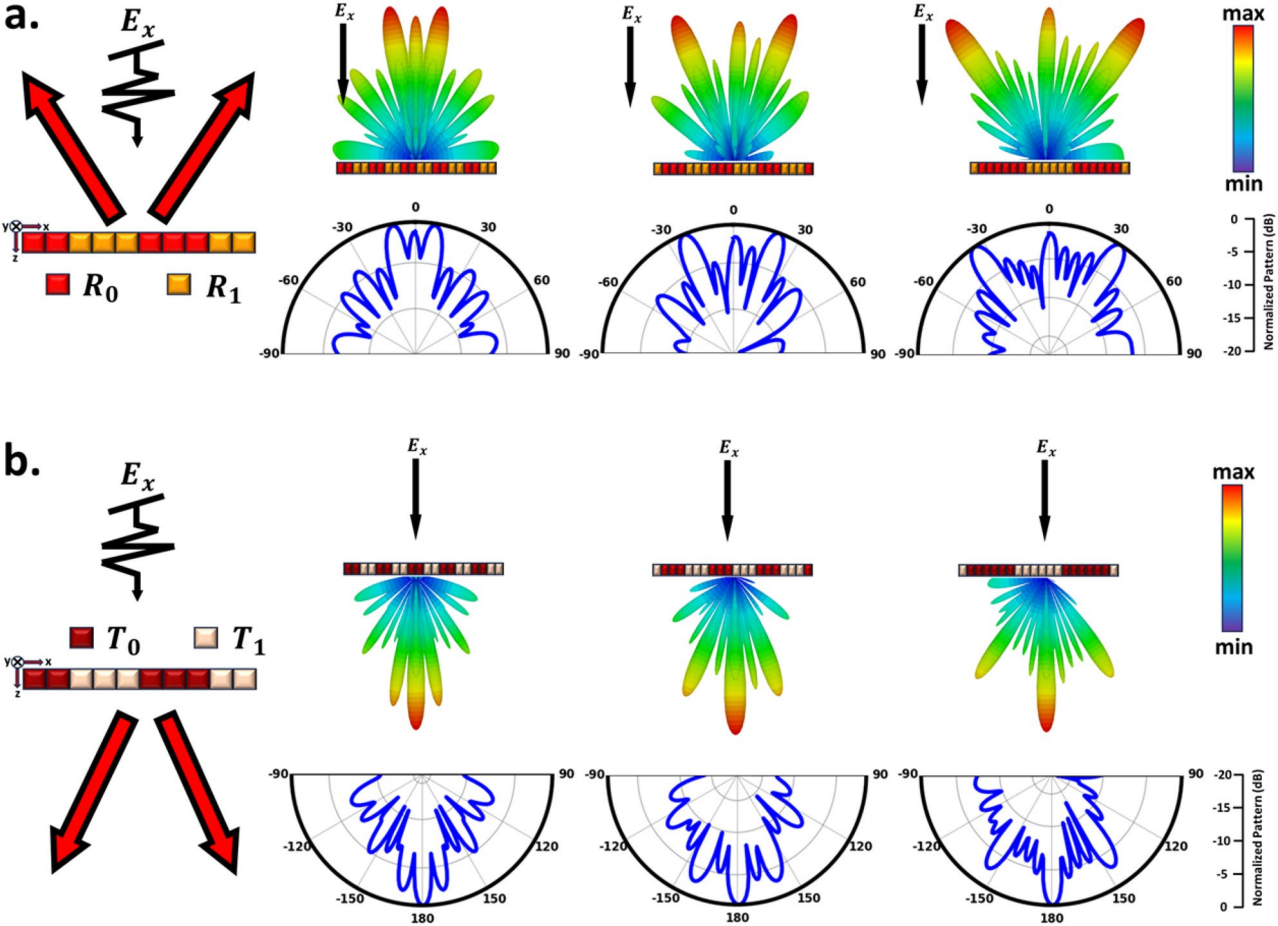
Simulation outcomes in reflection and transmission modes. (**a**) Simulation results for the reflection mode using coding sequences “$R_0R_0R_1R_1$...”, “$R_0R_0R_0R_1R_1R_1$...”, and “$R_0R_0R_0R_0R_0R_0R_1R_1R_1R_1R_1R_1$...” intended to generate twin beams at ±11^∘^, ±22^∘^, and ±34^∘^ in the backward half-space, respectively. (**b**) Simulation results for the transmission mode using coding sequences “$T_0T_0T_1T_1$...”, “$T_0T_0T_0T_1T_1T_1$...”, and “$T_0T_0T_0T_0T_0T_0T_1T_1T_1T_1T_1T_1$...” intended to generate twin beams at ±11^∘^, ±22^∘^, and ±34^∘^ in the forward half-space, respectively.

**Figure 4:**
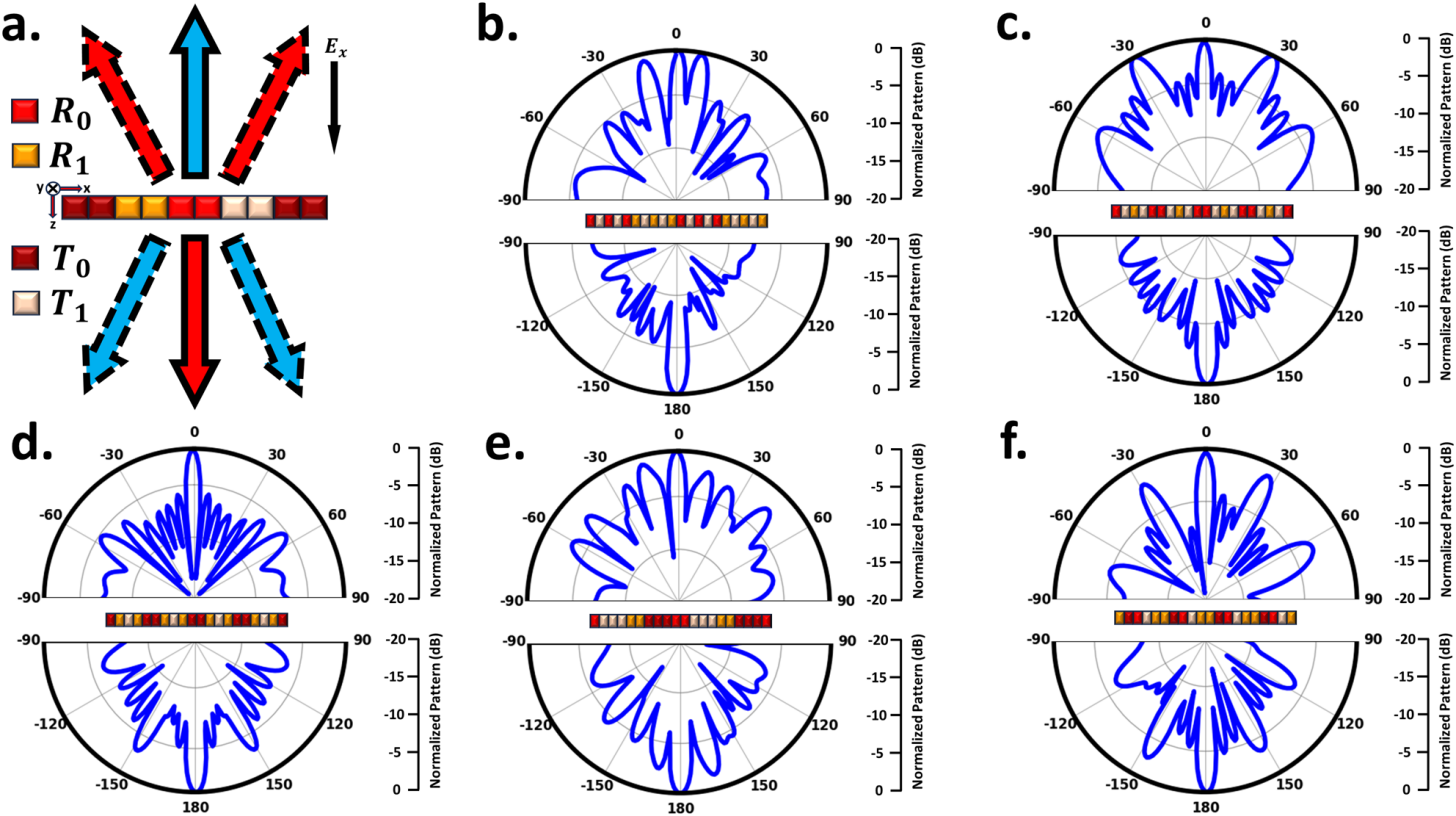
Illustrative schematic and simulated results for simultaneous 1-bit reflection/transmission Mode. (**a**) Schematic outlining the design concept. The far-field pattern of the full simulated metasurface space, which operates concurrently in reflection and transmission modes, with the coding sequence (**b**) “$R_0T_1R_0T_1R_1T_1R_1T_1R_1$...”, (**c**) “$R_0T_1R_1T_1R_0$...”, (**d**) “$T_0R_1T_1R_1T_0$...”, (**e**) “$R_0T_1T_1T_1R_1R_1T_0T_0T_0R_0$...”, and (**f**) “$R_1T_0R_0T_1R_0$...”.

6 $$\begin{aligned} \theta = \pm arcsin(\frac{\lambda _0}{2\pi } \frac{\Delta \phi }{D}) \end{aligned}$$

Where $\lambda _0$ is the wavelength at the central frequency, $\Delta \phi = \pi$ represents the phase difference between lattice, and D = MP (M is the number of meta-atoms in each lattice). As the design examples, we employ three distinct periodic coding sequences along the x-direction within the metasurface and then analyze their resulting reflection scattering patterns. These three sequences are obtained for M = 2, 3, and 6. Hence, the metasurface is theoretically anticipated to deflect the incident x-polarized wave in the reflection mode toward anomalous angles of $\theta$ = ± 11^∘^, ± 22^∘^, and ± 34^∘^ within the x–z plane, according to the generalized Snell’s law. To verify the preceding discussion, Fig.3a displays the simulated scattering patterns in the reference $\phi$ =0^∘^^∘^plane at f = 2.61 THz. The reflected beams split into twin beams symmetrically at the predesigned deviation angles $\theta$ = ± 11^∘^, ± 22^∘^, and ± 34^∘^, demonstrating a good agreement with theoretical predictions. This affirms the dynamic control of dual beams through the the Eq. 6 in the reflection mode. In the transmission state, the chemical potential of the reflection-transmission layer’s graphene is equal to 0 eV. In this state, wavefront manipulation is achieved by appropriately biasing the phase layer, effectively encompassing the front half-space. Adopting an approach analogous to that utilized in the reflection mode, we can partition the scattered wave into two symmetric transmission beams, thereby demonstrating an unconventional reflection behavior. Achieving this involves implementing a stripe coding pattern along the x-direction on the phase control layer. Three separate periodic coding sequences are employed for M = 2, 3, and 6 to dynamically control the angles of dual symmetrical beams. This dynamic control of the twin beam is realized through the manipulation of the chemical potential of the phase layers and employing stripe coding patterns along the x-direction. These patterns exhibit a phase difference of 180^∘^ and consist of “0” and “1” coding modes. Here, we denote these two states as the coding bytes “$T_0$” and “$T_1$”. As shown in Fig.3(b), in the transmission mode, the metasurface deflects the x-polarized wave towards anomalous angles of $\theta$ = ± 11^∘^, ± 22^∘^, and ± 34^∘^ within the x–z plane. Notably, these results not only align with the forecasted results from the Eq. 6 but also correspond to the observed functionality in the reflection mode, albeit within a different half-space arrangement. It’s important to acknowledge that the beam arising in the zeroth (middle) mode for both reflection and transmission modes is a consequence of the non-ideality of the full structure (limited number of supercells in the x-direction and unit cells in the y-direction).

**Figure 5:**
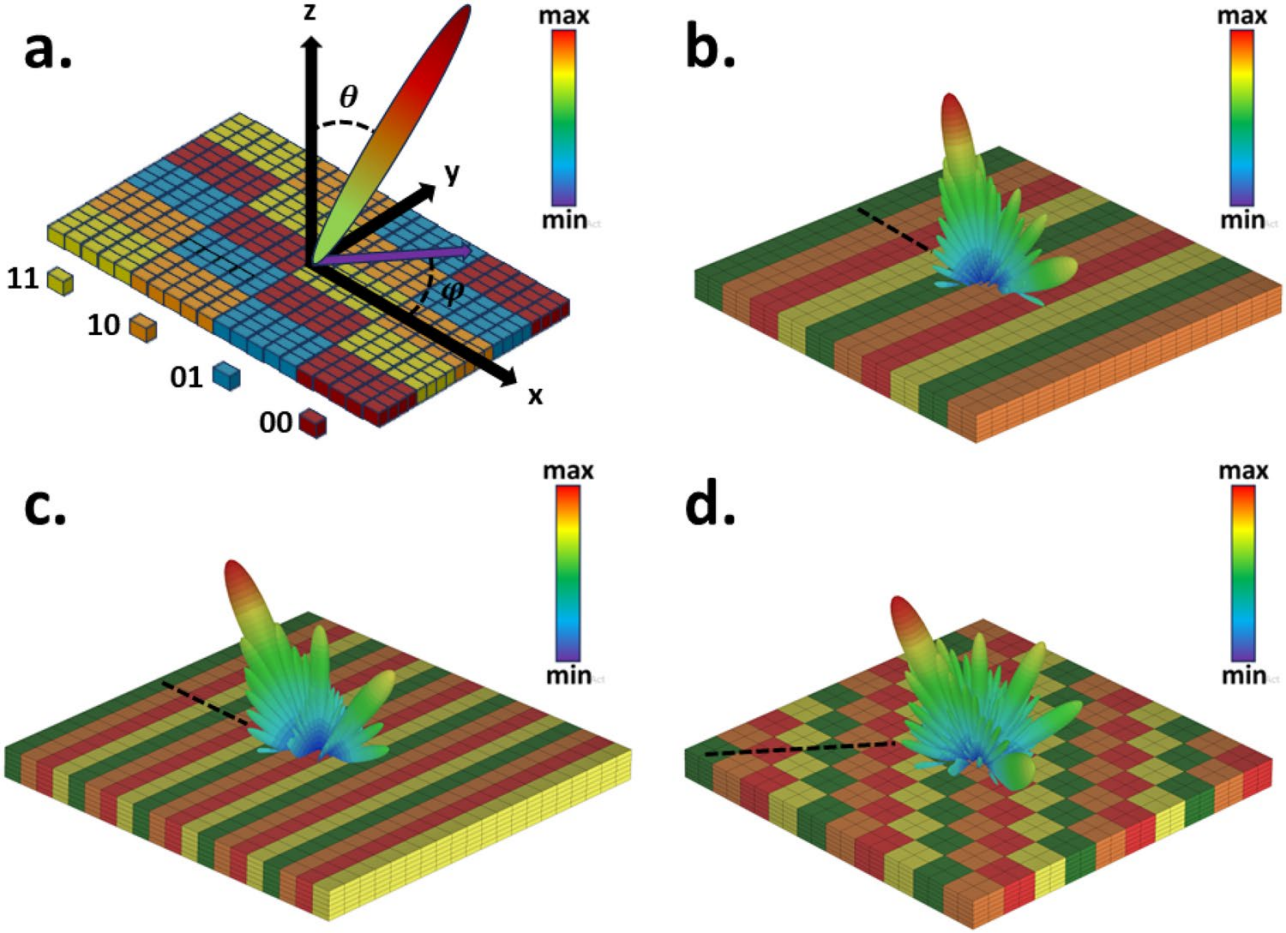
Full-wave simulation results of 3D scattering patterns in 2-bit reflection mode for demonstration of the anomalous reflection into an arbitrary pre-determined direction. (**a**) The proposed metasurface is suitably programmed with different switchable gradient coding sequences (00 01 10 11 ...) varying along both vertical and horizontal directions. (**b**) $\theta$ = 16.5^∘^, $\phi$ = 180^∘^, (**c**) $\theta$ = 35^∘^, $\phi$ = 180^∘^ and (**d**) $\theta$ = 35^∘^, $\phi$ = 225^∘^.

To achieve wave control full space, we simultaneously put the metasurface in both reflection mode (backward half-space) and transmission mode (forward half-space). As illustrated in Fig. 4, to achieve this objective, we position the lattice associated with reflection and transmission modes adjacent to each other in a striped manner. It’s important to highlight that for various applications requiring simultaneous wave control across the full-space, we incorporate 1-bit unit cells in the structure for both reflection and transmission modes. Through Phase calculation, quantization and finally optimization, we have determined the final layout, wherein the number of unit cells in each supercell, arranged in stripes, varies for both reflection and transmission modes to accommodate different modes effectively. Figure 4a shows the general schematic of the design for the multifunctional metasurface for the phase distribution in two modes of reflection and transmission. Through simultaneous manipulation of reflection and transmission, we can attain identical or distinct functionalities, exemplified by the control of dual beams in reflection mode-direct propagation transmission mode (indicated by red arrows), direct propagation reflection mode-control of dual beams in transmission mode (indicated by blue arrows), and control of dual beams in reflection mode-control of dual beams in transmission mode within two distinct half-spaces (indicated by red and blue dashed arrows). In the scenario of controlling dual beams in reflection mode-direct propagation transmission, the generation of reflective twin beams is attainable by arranging the digital states “$R_0$” and “$R_1$” in a striped coding pattern, while the direct transmission mode is realized by employing lattices with identical phases (“$T_1$”). As depicted in Fig. 4b, the striped arrangement of lattices with a 180^∘^ phase difference in the reflection mode results in the transformation of the reflected wave into two symmetrical beams with an angle of $\theta$ = ± 14^∘^, as previously described. On the other hand, in the transmission mode, where there is no phase difference between the lattices, the incident wave passes through without altering its direction. Through precise manipulation of lattice parameters and arrangement, the angle of the two beams can be effectively modulated to $\theta$ = ± 28^∘^ within the reflection mode. Remarkably, this alteration occurs while preserving the unaltered wave performance within the transmission mode, as demonstrated in Fig. 4c. Illustrated in Fig. 4d, interchanging the coding patterns between reflection and transmission modes induces alterations in the beam patterns across both the backward and forward half-space ultimately yielding direct propagation reflection mode-dual beam control in transmission mode. Evidently, in the reflection mode, the incident wave is reflected without directional alteration, while in the transmission mode is achieved symmetrical twin beams. In the dual beam control mode that encompasses both the backward and forward half-spaces, simultaneous manipulation is achieved through stripe arrangement (where lattices maintain a phase difference of 180^∘^) applied to both reflection and transmission modes. This configuration enables the concurrent control of dual beams across both spatial halves. Illustrated in Fig. 4e,f are the simulated outcomes demonstrating the generation of dual beams at $\theta$ = ± 14^∘^ and ± 28^∘^ angles for both reflection and transmission modes. Furthermore, with the modification of supercell dimensions, diverse angles can be attained, facilitating the control of both spatial halves. This methodology enables the dynamic manipulation of waves across the forward and backward spaces, achieved through the appropriate arrangement and distribution of phase layers and the reflection-transmission control layer.

**Figure 6:**
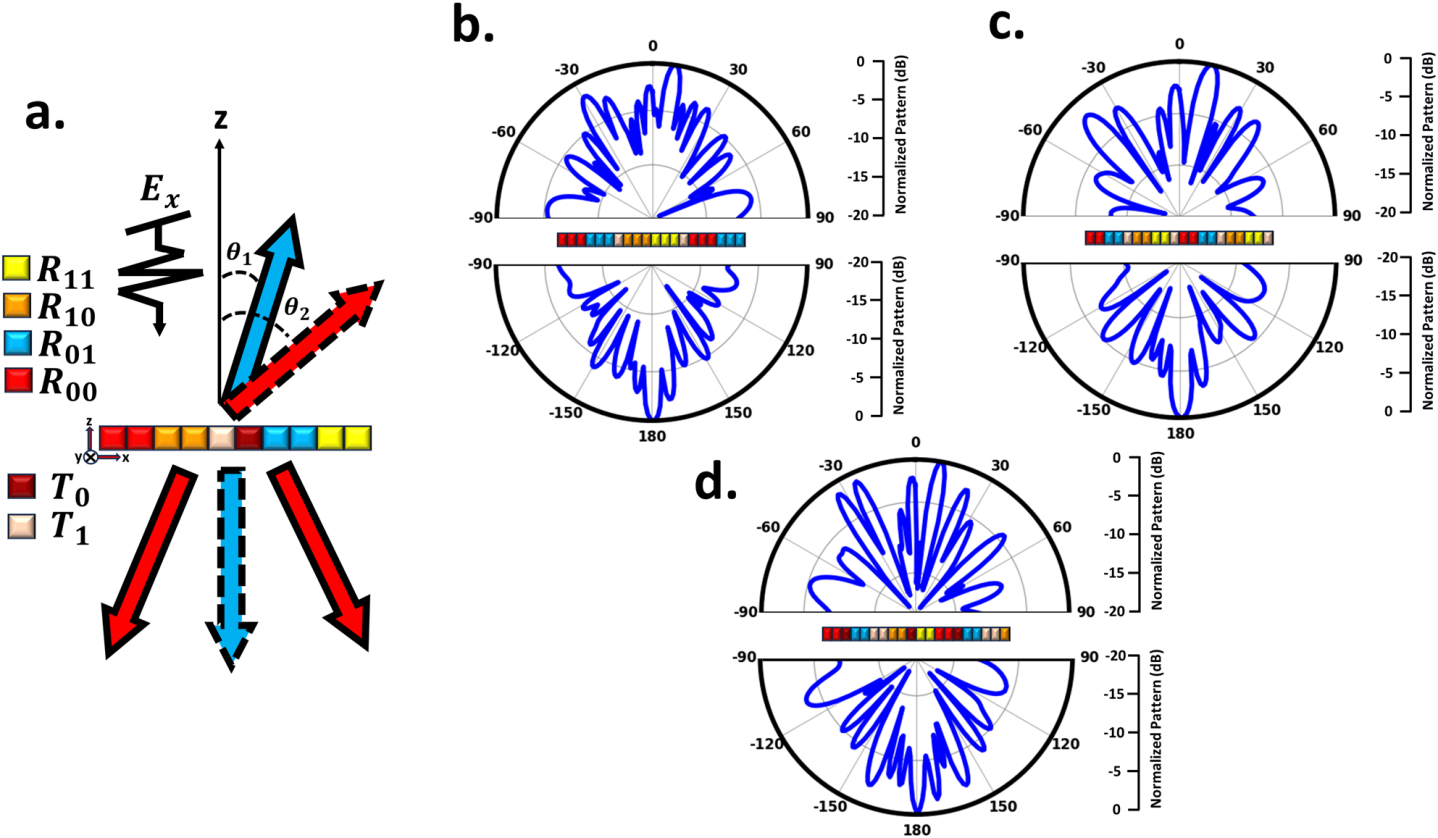
Illustrative schematic and simulated results for simultaneous 2-bit reflection/transmission mode. (**a**) Schematic outlining the design concept. The far-field pattern of the full simulated metasurface space, which operates concurrently in reflection and transmission modes, with the coding sequence (**b**) “$R_{00}R_{00}R_{00}R_{01}R_{01}R_{01}T_1R_{10}R_{10}R_{10}R_{11}R_{11}R_{11}T_1$...”, (**c**) “$R_{00}R_{00}R_{01}R_{01}T_1R_{10}R_{10}R_{11}R_{11}$...”, and (**d**) “$R_{00}R_{00}T_0R_{01}R_{01}T_1T_1R_{10}R_{10}R_{10}R_{10}T_0R_{11}R_{11}$...”.

#### Performance of the reconfigurable metasurface in 2-bit reflection and 1-bit transmission modes

Additionally, owing to the 2-bit (00, 01, 10, 11) phase control in the reflection mode, we explore the generation of reflected beams with arbitrary $\theta$ and $\phi$ angles with the appropriate phase control through the phase control layer. It’s crucial to highlight that manipulating reflected or transmitted beams using a 1-bit coding metasurface is not achievable^27^. Phase gradient encoding sequences fabricate synthetic surfaces with the capacity to establish predetermined in-plane wave vectors, thereby allowing precise control over the orientation of the reflected wavefront. Leveraging the lateral phase gradient, a suitably engineered transverse phase discontinuity profile implemented on the metasurface can adeptly steer the incident wave along a new predefined trajectory^36^. according to the generalized Snell’s law, the direction of the main rays ($\theta _m$, $\phi _m$) can be written as follows^77^:

7 $$\begin{aligned} sin\theta _{m} = \frac{\lambda _0}{2\pi }[(\frac{\Delta \phi _{x}}{D_{x}})^2+(\frac{\Delta \phi _{y}}{D_{y}})^2]^{\frac{1}{2}} \end{aligned}$$

8 $$\begin{aligned} tan\phi _{m} = \frac{\Delta \phi _{y}}{\Delta \phi _{x}}\frac{D_{x}}{D_{y}} \end{aligned}$$

Here, $\Delta \phi _{y}$ and $\Delta \phi _{x}$ are the phase differences of lattices along the y and x-directions, respectively and $D_y$ = $D_x$ = MP. Clearly, through design of the coding sequences, it becomes possible to directed the reflected beam to any desired pre-defined angle within each of the four quadrants in the backward half-space. Phase gradient encoding sequences denoted as “$R_{00}$”, “$R_{01}$”, “$R_{10}$”, “$R_{11}$” are designed in both the horizontal and vertical directions to achieve the desired $\theta _m$ and $\phi _m$ angles as depicted in Fig. 5a. To explore the redirection of the reflected wave to a predefined target angle, as can be seen from Fig. 5b, we employ $\Delta \phi _{x} = \pi /2$ and $\Delta \phi _{y}$ = 0^∘^-form phase gradient encoding sequences. These sequences are utilized to reflect radiation rays at specific angles, namely $\theta _m$ = 16.5^∘^ and $\phi _m$ = 180^∘^, while the value of M=2. By reducing (increasing) the value of M, the $\theta$ angle can be increased (decreased). As illustrated in Fig. 5c, when M=1, the radiation wave is reflected at an angle of $\theta _m$ = 35^∘^ and $\phi _m$ = 180^∘^. In the phase distribution depicted in Fig. 5d, the phase gradient’s coding sequence is designed in a manner that $\Delta \phi _{x} = -\pi /2$ and $\Delta \phi _{y} = \pi /2$. Consequently, this phase distribution allows for the reflection of the radiation wave in the direction corresponding to $\theta _m$ = 35^∘^ and $\phi _m$ = 225^∘^. It’s worth noting that our experimental results align exceptionally well with the corresponding theoretical expectations, demonstrating the accuracy of our approach. By applying an external DC voltage to the graphenes within the phase control (G2) section, controlled by the FPGA, instant access to a single beam at any desired angle in the reflection mode becomes possible.

Just like in Fig. 4, to attain full-space control, we position the meta-atoms responsible for both reflection and transmission modes simultaneously in stripes. However, there is a distinction: in the reflection mode, we utilize 2-bit unit cells instead of 1-bit units cells. Once more, employing employing Phase calculation, quantization and finally optimization, we derive the ultimate layout detailing the number of unit cells in each supercell and place them in stripes. This deliberate setup, in combination with the generation of an appropriate phase distribution, enables us to manipulate the wavefront in two half-spaces, offering a wide range of functions. These functions encompass not just beam steering in the reflection mode-direct propagation in the transmission mode but also beam steering in the reflection mode-controlling dual beams in the transmission mode. Fig. 6a illustrates the overall design schematic for the multi-purpose metasurface, featuring a 2-bit phase distribution in the reflection mode and 1-bit in the transmission mode. Through the simultaneous manipulation of reflection and transmission, various functions are achievable. These include beam steering at a specific angle, denoted as $\theta _1$, in the reflection mode-direct propagation in the transmission mode (indicated by blue arrows), beam steering at a specific angle, denoted as $\theta _2$, in the reflection mode-direct propagation in the transmission mode (indicated by red and blue dashed arrows), and beam steering at a specific angle, denoted as $\theta _2$, in the reflection mode-control of dual beams in the transmission mode (indicated by red arrows). In the scenario of beam steering reflective mode-the direct propagation transmission, control of a single beam within the angles $\theta _1$ or $\theta _2$, while phi=180^∘^, can be achieved by configuring the digital modes “$R_{00}$”,“$R_{01}$”, “$R_{10}$” and “$R_{11}$” in a striped coding pattern. Furthermore, the direct transmission mode is established through networks featuring identical phases denoted as “$T_{1}$”.Observing Fig. 6b and c, it becomes evident that accomplishing the concurrent beam steering in reflection mode-direct propagation in transmission mode can be attained by arranging the reflection and transmission elements adjacently. Attaining the desired $\theta _1$ and $\theta _2$ angles can be accomplished through adjustments to the meta-atoms count within the lattice. In reflection mode, organizing lattices into adjacent stripes with a 90^∘^ phase offset enables precise control of individual beams. Moreover, when transmission-mode lattices (all composed of $T_1$) are placed alongside reflection mode lattices, we not only achieve wavefront control in the reflection mode but also enable direct wave transmission. It’s worth noting that in Fig. 6b, where M=3, and in Fig. 6c, where M=2, we effectively steered the reflected waves at angles of $\theta _1$ =11^∘^ and $\theta _2$ =16^∘^, respective. Beyond achieving beam steering in the reflection mode-direct propagation in the transmission mode, we can also gain control over dual beams in the transmission mode. This is accomplished through the precise arrangement of each lattice in transmission mode, where there exists a 180^∘^ phase difference between them (T0 and T1), as illustrated in Fig. 6d.

## Possible fabrication method

The sample fabrication process can be outlined as follows, presenting a feasible approach within the scope of current fabrication technologies. Initially, a Quartz layer can be coated onto a silicon wafer using a spin-coating solution. Secondly, to facilitate the growth of ultrathin, high-quality SiO_2_ tunnel oxide, the p-Si wafers were introduced into a Rapid Thermal Oxidation (AS-One) chamber at 25 ^∘^C under a N2 flow rate of approximately 800 sccm. Subsequently, the temperature was raised to 900 ^∘^C (with a ramp up rate of about 25 ^∘^C/s), and maintained for SiO_2_ growth with an oxygen flow rate of around 800 sccm at 900 ^∘^C for 90 seconds. Finally, the temperature was gradually decreased from 900 to 25 ^∘^C at a rate of about 3 ^∘^C/s under a N2 flow rate of approximately 800 sccm. Graphene samples can be produced by utilizing chemical vapor deposition (CVD) graphene grown on copper foil. After being transferred to SiO_2_ /poly-Si wafers, the excess graphene was removed in the sheets using 100 keV electron beam lithography in PMMA followed by an oxygen plasma etch, leaving only the square or ribbons shaped regions. A 20 nm thick layer of Al_2_O_3_ was then deposited using atomic layer deposition (ALD), with the growth of Al_2_O_3_ achieved through the utilization of trimethyl aluminum (TMA) and H_2_O as precursors at a temperature of 200 ^∘^C^86–88^. Lastly, a quartz layer is once more applied onto a silicon wafer using a spin coating solution and positioned atop the Al_2_O_3_ layer. This process is iterated five additional times, with each layer sequentially stacked atop the other^84,89,90^.

## Discussion

In summary, for the first time, we have designed a graphene-assisted reprogrammable intelligent metasurface in the THz band, which offers an unprecedented ability to independently and simultaneously manipulate EM waves in real time in both half-spaces. The proposed metasurface possesses the unique capability to independently and simultaneously control wavefronts in both reflection and transmission modes, all within the same polarization and frequency channel. The meta-atom design incorporates two graphene sections. Through the control of each graphene section’s chemical potential using an external electronic source, we can manipulate the EM wave to serve various functions. In addition to real-time amplitude control for both reflection and transmission modes, we can also harness graphene to manage the phase of the EM wave. Such capabilities enable us to achieve diverse functionalities, including controlling beam steering and two beams in reflection mode, control of two beam in transmission mode, and combining functionalities from both reflection and transmission modes. The proposed metasurface is expected to have wide applications in such as encryption, miniaturized systems, and next-generation wireless intelligent communications.

### Supplementary Information

Supplementary Information.

## Data Availability

The data that support the findings of this study are available from M.S. but restrictions apply to the availability of these data, which were used under license for the current study, and so are not publicly available. Data are however available from the authors upon reasonable request and with permission of M.S. (email: soleimani@iust.ac.ir).
