# Supplementary material for: Reprogrammable reflection-transmission integrated coding metasurface for real-time terahertz wavefront manipulations in full-space
Source: Sci Rep. 2024 May 15;14:11156. doi: 10.1038/s41598-024-61638-7 (PMC11096410; doi:10.1038/s41598-024-61638-7)
Supplement: Supplementary file 1 — Supplementary Information. [file 41598_2024_61638_MOESM1_ESM.docx]

**Supplementary Information for**

**Reprogrammable Reflection Transmission Integrated Coding Metasurface for Real Time Terahertz Wavefront Manipulations in Full Space**

**Parsa Farzin**^1^**, AmirSaman Nooramin**^1^**, and Mohammad soleimani**^1,^*

1 School of Electrical Engineering, Iran University of Science and Technology, Tehran, 1684613114, Iran

*soleimani@iust.ac.ir

**The Supplementary file includes:**

The detailed explanations of A) External biasing Architecture; B) Effects of each layer; C) The circuit model of meta-atoms.

**Supplementary Appendix A (External biasing Architecture)**

The complex surface conductivity of graphene is closely tied to the concentration of free carriers, denoted as “n” (cm^-2^). This relationship is defined by the following formula, which connects n to the chemical potential of graphene [1].

| $\mu_{c}=\pm\hbar v_{f}\sqrt{\left( \pi\left\vert n_{g} \right\vert\right)}$ | (s1) |
| --- | --- |

Where $v_{f}=0.9\times{10}^{6} m.s^{-1}$ is the Fermi velocity. The net carrier density (n) can be dynamically modulated by applying a direct current (DC) voltage between the graphene and the silicon surface, thereby facilitating the augmentation or reduction of charge carriers within the graphene layer as needed. In accordance with the Fowler-Nordheim tunneling mechanism [2], the graphene charge density can be expressed as the integral of the tunneling current $J_{\mathrm{SiO}_{2}}$ in the $\mathrm{SiO}_{2}$, which is given by:

| $n_{g}=\frac{1}{q}\int_{0}^{t_{0}} J_{{SiO}_{2}}dt$  $J_{{SiO}_{2}}= \frac{q^{3}}{16\pi^{2}\hbar\varphi_{{SiO}_{2}}}E_{{SiO}_{2}}^{2}\exp\left( -\frac{4\sqrt{2m}\varphi_{{SiO}_{2}}^{3/2}}{3\hbar qE_{{SiO}_{2}}} \right)$ | (s2)  (s3) |
| --- | --- |

where $t_{0}$ is the duration time of the voltage applied on the Si, $\varphi_{\mathrm{SiO}_{2}}=3.2 eV$ [3] is the barrier height of $\mathrm{SiO}_{2}$, m is effective mass of electron, and $E_{\mathrm{SiO}_{2}}$ shows the electric field intensity in $\mathrm{SiO}_{2}$. It is calculated by

| $E_{{SiO}_{2}}=\frac{V_{DC}}{h_{{SiO}_{2}}+{Al}_{2}O_{3}\left( \varepsilon_{r, SiO_{2}}+\varepsilon_{r,{Al}_{2}O_{3}} \right)}-\frac{qn}{\varepsilon_{r, SiO_{2}}\left[ 1+\left( \varepsilon_{r,{Al}_{2}O_{3}}h_{{SiO}_{2}} \right)/\varepsilon_{r, SiO_{2}}h_{{Al}_{2}O_{3}} \right]}$ | (s4) |
| --- | --- |

Where $h_{\mathrm{SiO}_{2}}=10 nm$ ($\varepsilon_{r, SiO_{2}}=3.9$) and $h_{\mathrm{Al}_{2}O_{3}}=20 nm$ ($\varepsilon_{r,\mathrm{Al}_{2}O_{3}}=9$) are the thickness (relative permittivity) of $\mathrm{SiO}_{2}$ and $\mathrm{Al}_{2}O_{3}$, respectively [4], and $V_{\mathrm{DC}}$ indicates the applied voltage to the graphene layer. The change of $n_{g}$ approximately follows $n_{g}= \varepsilon_{0}\varepsilon\Delta V/hq$in which $\varepsilon$ and $\varepsilon_{0}$ are the permittivities of $\mathrm{SiO}_{2}$ and free space, respectively and $\Delta V=V_{gate}-V_{DC}$ in which, $V_{gate}$ and $V_{DC}$ are the applied voltages to the gate connection and graphene surface, respectively [5]. Hence, by precisely tuning the electrostatic voltage bias applied to graphene, it becomes feasible to achieve the desired chemical potential, as described by the following expression:

| $\Delta V=\frac{qh\mu_{c}^{2}}{\pi\varepsilon_{0}\varepsilon_{r}\hbar^{2}v_{f}^{2}}$ | (s5) |
| --- | --- |

Given that the physical and dielectric properties of the metasurface remain unalterable post-fabrication, reconfigurability is exclusively achieved by adjusting the bias across all two graphene section. In pursuit of this objective, as can be seen from **Supplementary Figure S1,** we've integrated all graphene layers within the structure using the Si-$\mathrm{SiO}_{2}$-Graphene-$\mathrm{Al}_{2}O_{3}$-Si configuration. To modulate the Fermi energy of graphene and consequently regulate its surface conductivity, a set of three adjustable DC gate voltages ($V_{1}$: voltage releated to G1, and $V_{2}$: voltage releated to G2) are employed to manage each section of the graphene. the required DC voltage bias can be applied to each graphene-based digital meta-atom via a gold (Au) electrical contact where Au contact touches the top and bottom Si layers.

**Quartz**

**Gold**

**Graphene**


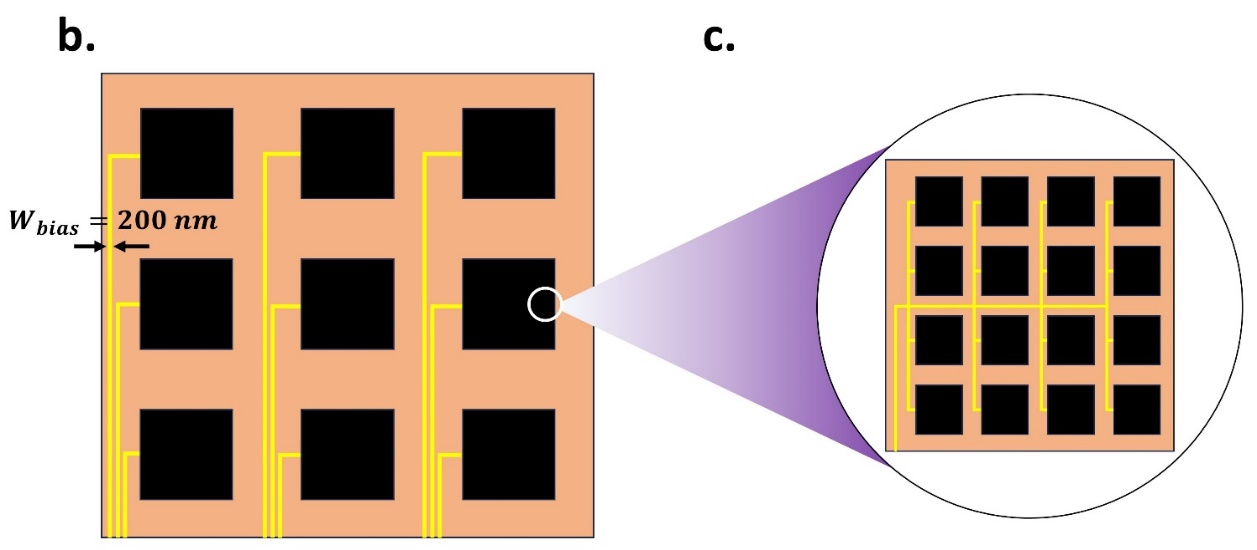

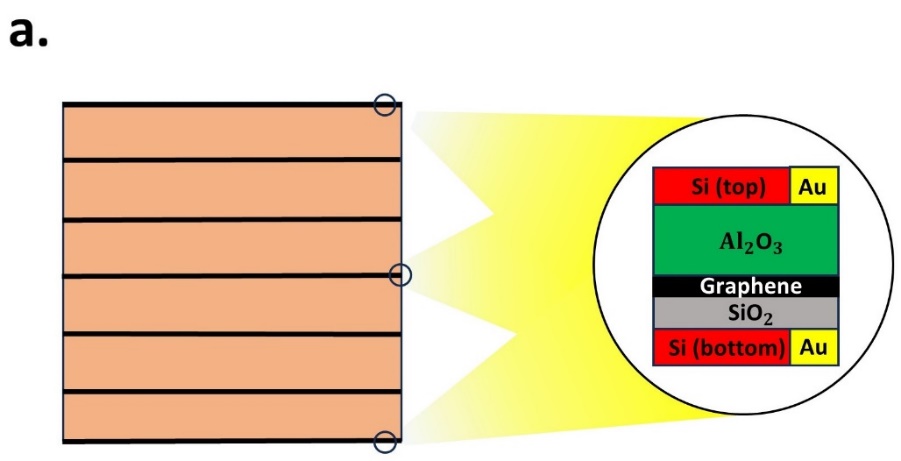


**Supplementary Figure S1.** (a) Layers required to bias graphene layers (b) Proposed metasurface (top view) (c) Meta-atoms powered by 2D bias networks with external DC voltages enabling simultaneous EM wave manipulation for varied functions.

**Supplementary Appendix B (Effects of each layer)**

To examine the influence of layer count on both reflection and transmission modes, we varied the phase control section from 1 layer to 4 layers. Figures (a)-(d) make it evident that, in all cases, achieving the desired phases in both reflection and transmission modes is unattainable when the layer count is less than 5.

| 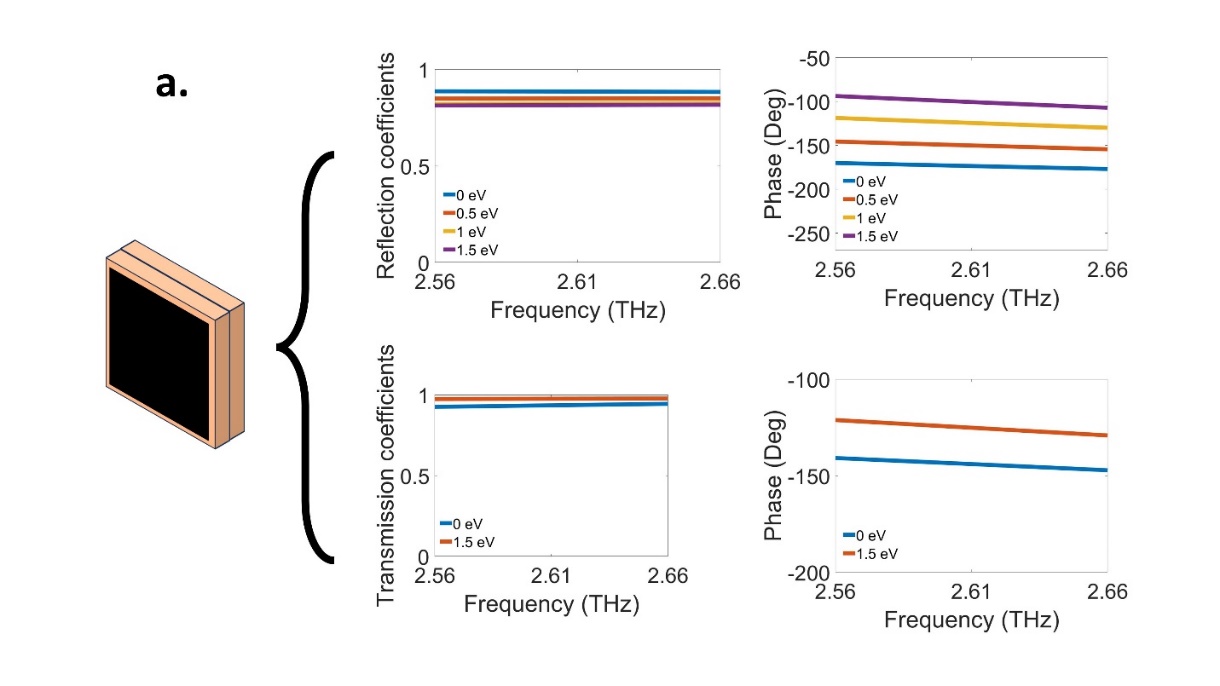  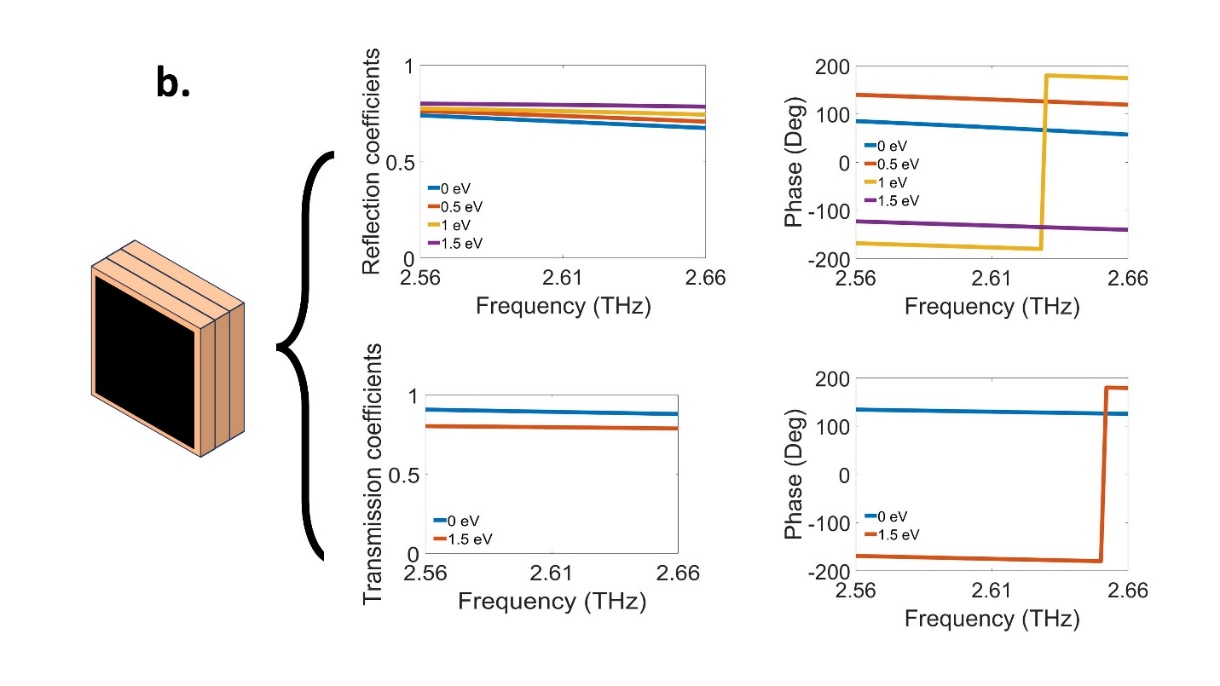 |
| --- |
|  |
| 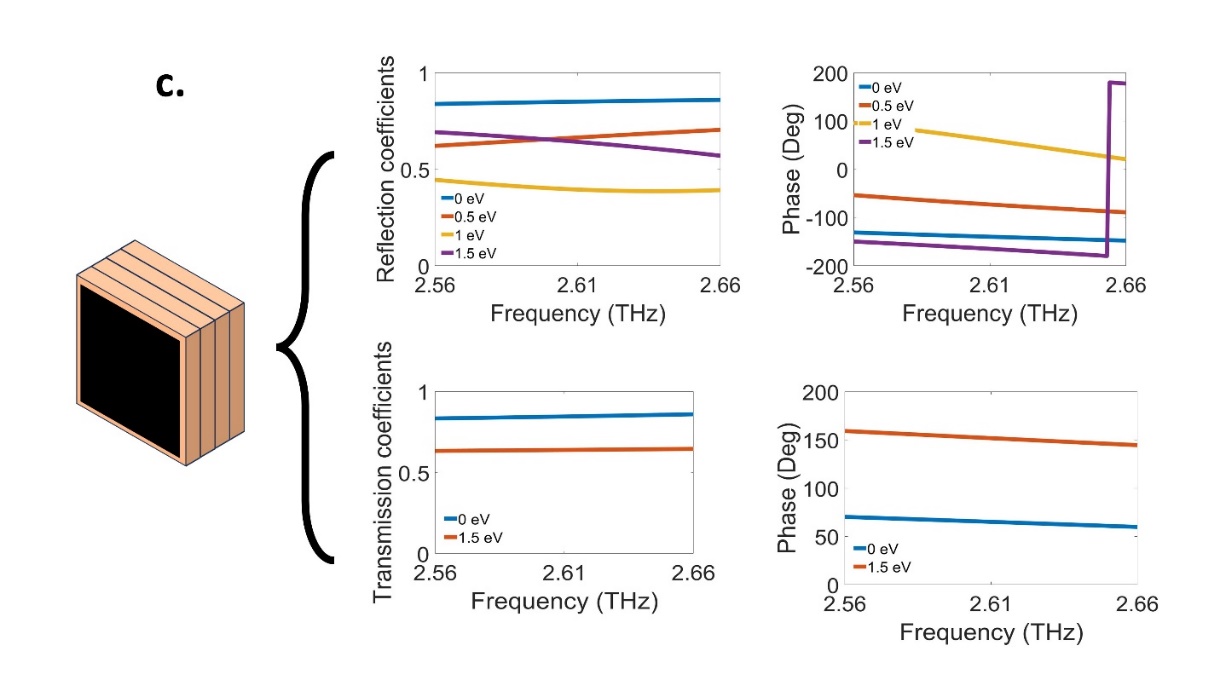 |
| 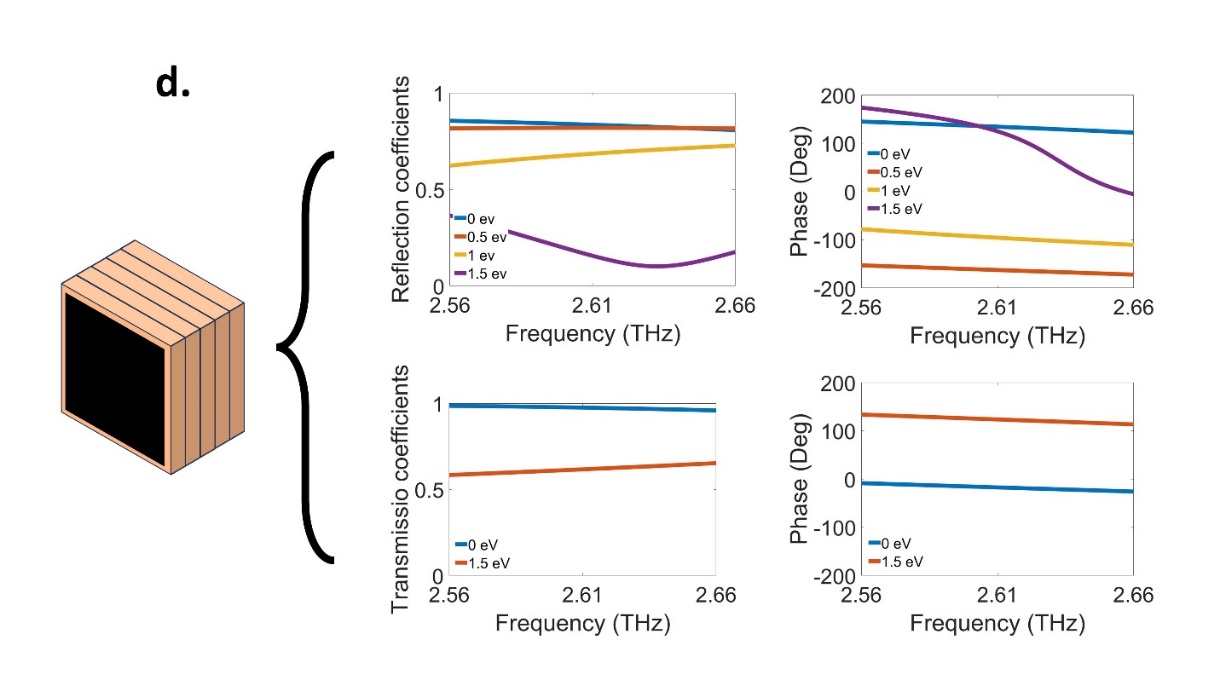 |

**Supplementary Figure S2.** The effect of the number of layers of the phase control section on the amplitude and phase of the reflected and transmitted wave. (a) 1-layer, (b) 2-layer, (c) 3-layer, and (d) 4-layer.

**Supplementary Appendix C (The circuit model of meta-atoms)**


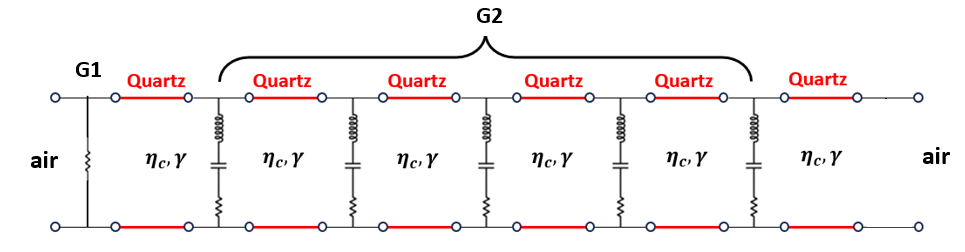


**Supplementary Figure S3.** The proposed circuit model for prediction of the reflection spectra of the employed meta-atoms. The parameters of η and γ represent the intrinsic impedance and the propagation constant of the substrates.

| 1. **R0:**   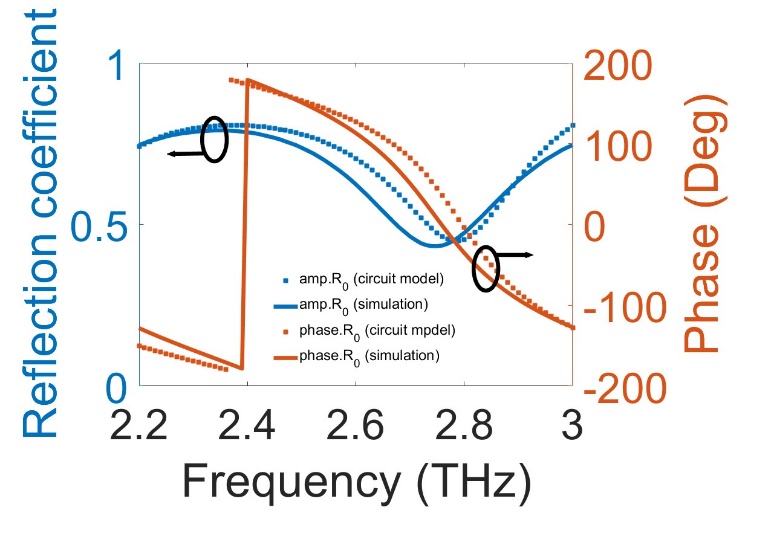 | 1. **T1:**   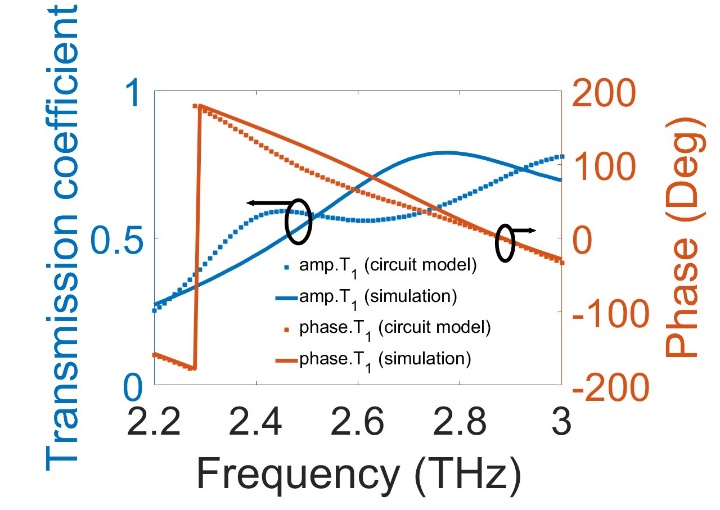 |
| --- | --- |

**Supplementary Figure S4.** A comparison between the full-wave simulations and the circuit model predictions of (a) amplitude and phase of R0, (b) amplitude and phase of T1.

**Supplementary Table S1.** The values of the circuit components for the employed graphene metasurfaces.

| **Function** | **section** | **R** (𝛀) | **L**(pH) | **C**(fF) |
| --- | --- | --- | --- | --- |
| Reflection(R01) | G1  G2 | 145  110 | -  40 | -  0.5 |
| Transmission (T1) | G1  G2 | 823  10 | -  12 | -  440 |

[1] Huang, Yuanyuan, et al. "Tunable circular polarization conversion and asymmetric transmission of planar chiral graphene-metamaterial in terahertz region." *Carbon* 119 (2017): 305-313.

[2] Lenzlinger, M., and E. H. Snow. "Fowler‐Nordheim tunneling into thermally grown SiO2." *Journal of Applied physics* 40.1 (1969): 278-283.

[3] Peng, Xi-Liang, et al. "An active absorber based on nonvolatile floating-gate graphene structure." *IEEE Transactions on Nanotechnology* 16.2 (2017): 189-195.

[4] Li, Yan, et al. "Graphene-based floating-gate nonvolatile optical switch." *IEEE Photonics Technology Letters* 28.3 (2015): 284-287.

[5] Novoselov, Kostya S., et al. "Electric field effect in atomically thin carbon films." *science* 306.5696 (2004): 666-669.
